# Automatic model for cervical cancer screening based on convolutional neural network: a retrospective, multicohort, multicenter study

**DOI:** 10.1186/s12935-020-01742-6

**Published:** 2021-01-07

**Authors:** Xiangyu Tan, Kexin Li, Jiucheng Zhang, Wenzhe Wang, Bian Wu, Jian Wu, Xiaoping Li, Xiaoyuan Huang

**Affiliations:** 1 Department of Obstetrics and Gynecology, Tongji Hospital, Tongji Medical College, Huazhong University of Science and Technology, 430030 Wuhan, Hubei China; 2 College of Computer Science & Technology, Zhejiang University, 310027 Hangzhou, China; 3 Data Science and AI Lab, WeDoctor Group Limited, 311200 Hangzhou, China; 4 School of Public Health, Zhejiang University, 310027 Hangzhou, China

**Keywords:** Cervical cancer, ThinPrep cytologic test (TCT), Deep leaning, Convolutional neural network (CNN)

## Abstract

**Background:**

The incidence rates of cervical cancer in developing countries have been steeply increasing while the medical resources for prevention, detection, and treatment are still quite limited. Computer-based deep learning methods can achieve high-accuracy fast cancer screening. Such methods can lead to early diagnosis, effective treatment, and hopefully successful prevention of cervical cancer. In this work, we seek to construct a robust deep convolutional neural network (DCNN) model that can assist pathologists in screening cervical cancer.

**Methods:**

ThinPrep cytologic test (TCT) images diagnosed by pathologists from many collaborating hospitals in different regions were collected. The images were divided into a training dataset (13,775 images), validation dataset (2301 images), and test dataset (408,030 images from 290 scanned copies) for training and effect evaluation of a faster region convolutional neural network (Faster R-CNN) system.

**Results:**

The sensitivity and specificity of the proposed cervical cancer screening system was 99.4 and 34.8%, respectively, with an area under the curve (AUC) of 0.67. The model could also distinguish between negative and positive cells. The sensitivity values of the atypical squamous cells of undetermined significance (ASCUS), the low-grade squamous intraepithelial lesion (LSIL), and the high-grade squamous intraepithelial lesions (HSIL) were 89.3, 71.5, and 73.9%, respectively. This system could quickly classify the images and generate a test report in about 3 minutes. Hence, the system can reduce the burden on the pathologists and saves them valuable time to analyze more complex cases.

**Conclusions:**

In our study, a CNN-based TCT cervical-cancer screening model was established through a retrospective study of multicenter TCT images. This model shows improved speed and accuracy for cervical cancer screening, and helps overcome the shortage of medical resources required for cervical cancer screening.

## Background

Cervical cancer is one of the most common malignant tumors in the world, and it is the fourth leading cause of cancer in women [1–3]. The morbidity and mortality of cervical cancer in the developing countries are distinctly higher than those in the developed countries [1, 4]. About four out of five medical cases occur in developing countries, and especially in China and India. Human papillomavirus (HPV) infections have been established as a main cause of cervical cancer [5–7]. Based on a survey of 30,207 cases at 13 Chinese medical centers, the HPV infection rate in China was found to be about 17.7% [8].

The incidence of cervical cancer can be reduced through early screening [9, 10]. For severe cervical cancer types, screening can help in avoiding cancer-related deaths [11]. In 2012, the National Comprehensive Cancer Network (NCCN) published clinical practice guidelines for cervical cancer screening. These guidelines show that the combination of HPV testing and cytology has been used as a primary option for cervical cancer screening in women [12].

In the United States, the number of deaths from cervical cancer has decreased since the implementation of widespread cervical cancer screening from 2.8 to 2.3 deaths per 100,000 women between 2000 and 2015 [13]. However, cervical cancer screening is still a problem with low diagnostic sensitivity and specificity, especially in developing countries. While cervical cancer goes undetected in some patients, it is overtreated in others [14]. In short, it is important to improve the efficiency and accuracy of cervical cancer screening, especially in China and other developing countries.

In the 1990 s, ThinPrep cytologic test (TCT) technology was approved by the Food and Drug Administration (FDA) for clinical applications. Compared with the pap smear, this technique reduced the effects of mucus, blood, and inflammation in cervical cancer screening. It can also effectively reduce the dissatisfaction rate of cell screening by maintaining or improving the sensitivity of disease screening [15–17]. According to Chinese guidelines for diagnosis and treatment of cervical cancer published in 2018, the recommended preliminary screening method is TCT combined with HPV screening [18].

The diagnostic results of the TCT were classified using the Bethesda system [19]. This system represents an artificial screening method for determining the precancerous cervical lesions by microscopic assessment of the changes in cytoplasm, nuclear shape, and the fluid base color [18]. According to the Bethesda system, the smears are mainly divided into the following categories: Intraepithelial Lesion/Malignant Lesion cell (NILM), atypical squamous cells of undetermined significance (ASCUS), low-grade squamous intraepithelial lesion (LSIL), and high-grade squamous intraepithelial lesion (HSIL). Above all, this method requires trained pathologists to make a correct diagnosis at the cellular recognition stage.

In developing countries with a large population, like China, the following three problems are mostly observed in cervical screening that reduces its diagnostic accuracy: (1) identification of cells on TCT smear images is subjective and mostly relies on the experience and technology of the pathologists. It has been reported that the increase in pathologists’ workload also leads to a reduced diagnostic sensitivity [20]. (2) The correct diagnosis of TCT images requires much of the pathologists’ time. However, there is a significant shortage of pathologists in China. Indeed, 68.4% of the pathologists who provide cervical cancer screening services have only primary technical qualifications or no qualifications at all. (3)The gap between urban and rural medically trained pathologists has been increasing [21]. Moreover, it takes more time and effort for human eyes to read images, the number of readings per day is also limited [22].

With the rapid development of science and technology, the emergence of artificial intelligence has provided more sensitive and effective solutions to many medical imaging problems [23, 24]. In recent years, deep learning algorithms have helped to identify patterns in classifying and quantifying medical images [25]. Significant progress has been made in the computer-aided diagnosis of medical images, such as the skin cancer diagnosis from melanoma images [25, 26] and the detection of pulmonary nodules via computerized tomography (CT) images [27, 28] and image-base detection of hepatocellular carcinoma using multiphasic magnetic resonance imaging (MRI) [29]. The concept of deep learning originated from the area of artificial neural network (ANN), which was helpful to extracting appropriate features from medical images. Among them, one of the leading neural networks for image recognition is a convolutional neural network (CNN) [30]. At present, this method is applied in most deep learning applications to achieve good detection results[31, 32].

We have developed a target recognition system based on a CNN model to assist pathologists in the diagnosis of early cervical cancer screening. This method is based on faster region convolutional neural networks (Faster R-CNN)[33] to locate and identify lesion cells in the cervical TCT images, extract features, and automatically optimize them through updating the convolutional kernel parameters in the cyclic training process [34]. Convolutional neural networks have the advantages of automatically learning features during training, and possessing strong inferential capabilities in comparison with conventional image processing methods in complex scenes [35, 36].

The trained faster RCNN model can automatically identify target regions and reduce the false detections of other non-target regions. As well, this model can generate region proposals on the feature map of the target region and its adjacent areas [33, 37]. The region proposal features can distinguish between target and non-target regions through merging layers and classifiers. It can learn the common characteristics of cells of the same lesion grade and the characteristic differences among different lesion grade cells. The identification of generalized performance was reported to be better than performance of conventional image processing methods under the conditions of cell deformation and cell superposition.

In this research project, we have successfully developed an auxiliary diagnosis model for the diagnosis of early cervical cancer. This model separated the positive or negative images effectively, minimizing the rate of missed diagnosis. It also reduced pathologists’ workload by saving their time and improved the accuracy of cervical cancer screening.

## Methods

**Fig. 1:**
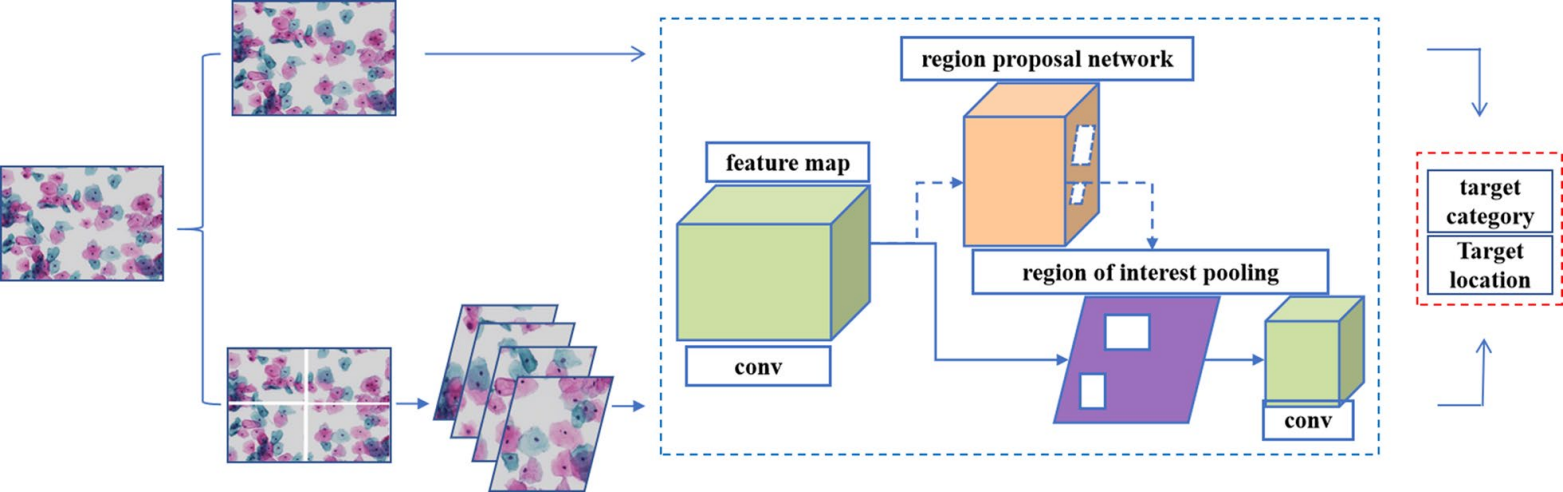
Framework of the proposed convolutional neural network (CNN) system for cervical cancer screening. The convolution network extracts the image information to obtain the feature map, and the proposal network screens the target region to obtain the target position information on the basis of the feature map. The pathological cells and their locations were identified by a convolutional classifier based on the feature map and target location. Pathological cell information was obtained by combining the recognition results of the two networks. The results of the two models are similar. However, the two structural models are trained with images of different magnification levels of 200X and 400X, and the parameters of the two models are different

The analysis pipeline for the detection of cervical cancer from the TCT smear using the Faster R-CNN is depicted in Fig. 1.

### Study design and participants

Images of TCT were obtained from multiple collaborating hospitals and research institutes in China. The collaborating entities are: (1) The First Affiliated Hospital, Sun Yat-sen University, (2) The First Affiliated Hospital, Xi’an Jiaotong University, (3) Shanghai General Hospital, Shanghai Jiaotong University, School of Medicine, (4) Shanghai First Maternity and Infant Hospital, (5) The First Affiliated Hospital of Soochow University, (6) Tongji Hospital, Tongji Medical College, Huazhong University of Science and Technology, (7) The Central Hospital of Wuhan, Tongji Medical College, Huazhong University of Science and Technology, (8) Xiangyang Hospital Affiliated to Hubei University of Medicine, and (9) the Molecular Department, Kindstar Global, Wuhan. About 16,000 TCT images of cervical brush smears were randomly selected and collected from outpatients at the above nine hospitals. All TCT images were histopathologically diagnosed as with different grades of a lesion by at least two local experienced pathologists according to the Bethesda system. This diagnosis was used as the gold standard for subsequent experiments. The images were sent to Tongji Hospital affiliated to Tongji Medical College of Huazhong University of Science and Technology, and the abnormal cells were examined and labeled by a pathologist.

### Image preparation and preprocessing

A pathologist from Tongji Hospital examined and preprocessed all images. High-quality images that met the requirements were selected. The inclusion criteria were as follows: The cells were evenly spread on the slide, the field of vision was clear, and the number of cells in the slide were moderate, with fewer overlapping cells.

**Fig. 2:**
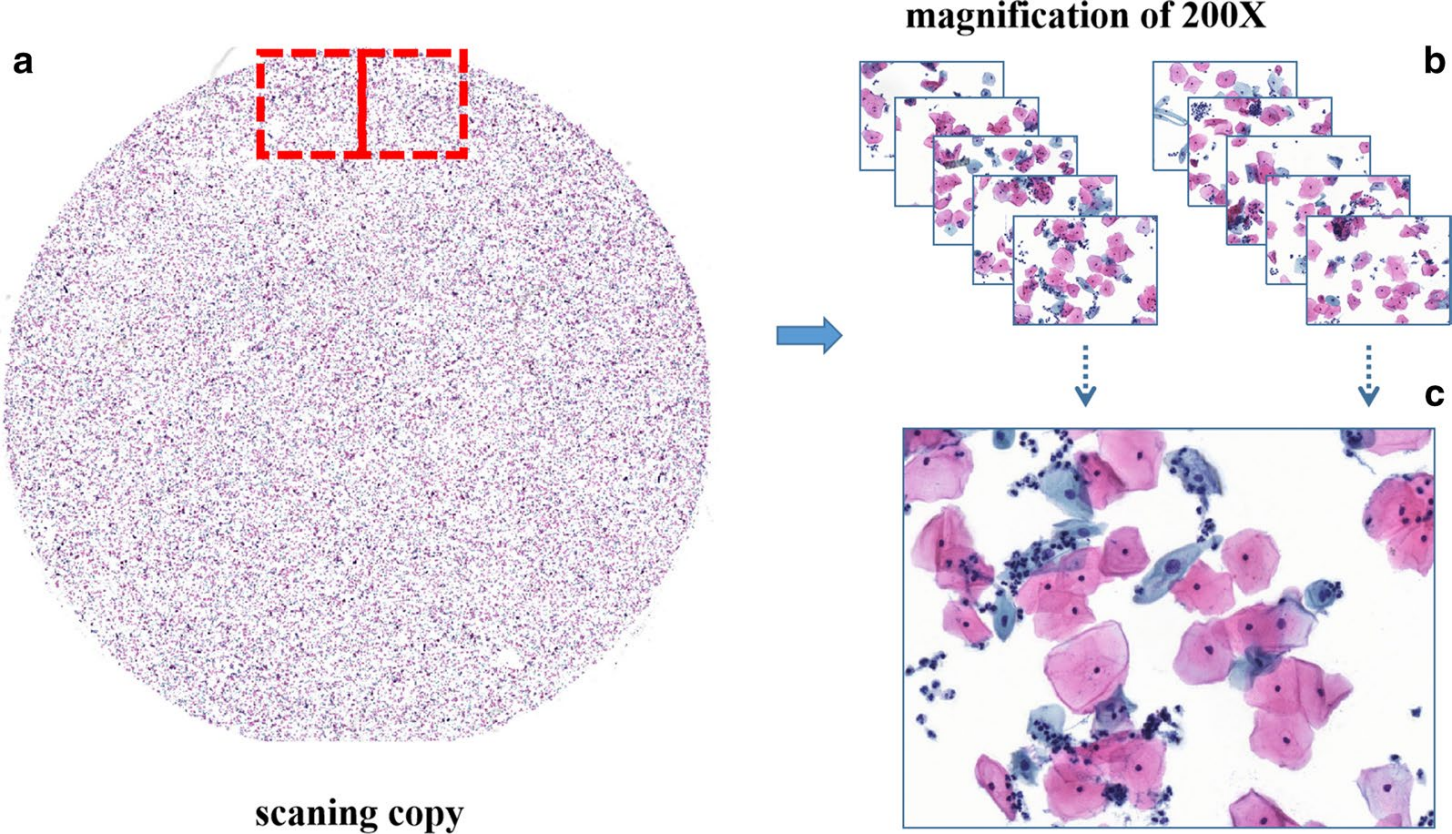
An overview of the seamless slider technique. Use the seamless slider technology to segment non-JPG images or large scanned image parts at a 200X magnification level. The whole ThinPrep cytologic test (TCT) smear image in **a** was divided into a large number of single-field images as shown in **b**. For a sample of image details, a random image from **b** is enlarged as shown in **c**

Images of non-JPG formats (such as scanned copies from slides) couldn’t be directly marked by pathologists because of the extra-large image size. So, these images were sent to Zhejiang University Rui Medical Artificial Intelligence Research Center for further processing and segmentation. One slide image was divided into 1407 single-field images with enlarged cervical cytology images using seamless slider technology. Figure 2 displays the process of the seamless slider technique, with a scanned image size of 83,712 × 86,784 × 3. In this work, the eyepiece has a 10X magnification, while the objective lens has magnifications of 20X or 40X. Hence, images with combined magnification factors of 200X and 400X were obtained. Then, those images were sent to the Tongji Hospital for creating test data through labeling irregular cells in images. The LabelImg software (Version 1.5.0.), an open-source graphical image annotation tool, was used to annotate the lesion areas in the eligible images. The primary purpose of lesion labeling was to train a model to recognize the abnormal cells. After that, all images with abnormal cell labeling were sent to Zhejiang University Rui Medical Artificial Intelligence Research Center for model training.

### Image processing

#### Image patching

Cervical cytology has a more diverse form of color expression compared with the standard red and blue. Because of the differences in the staining methods and the severity of cervical cancer, the cells show varied and complicated cell morphology patterns. The image analysis flow chart for the Faster R-CNN system for the detection of cervical cancer from the TCT smear is depicted in Fig. 1. In this paper, we created three different datasets. The first one was named as the training dataset with 13,775 images, of which 5414, 4292, and 4069 were ASCUS, LSIL, and HSIL images, respectively. These images showed 200X and 400X magnification levels. We used this dataset to train the Faster R-CNN model. The second dataset was named as the validation dataset with 2301 images, of which 1000 images had a 200X magnification and 1301 images had a 400X magnification. The detailed statistics of each kind of picture scan piece or field of view are presented in Table 1.

**Table 1:** Details of sample sizes of the three datasets including training dataset, validation dataset, and test dataset

A: Training dataset				
	ASCUS	LSIL	HSIL	Total
Images	5414	4292	4069	13775
B: Validation dataset				
	ASCUS	LSIL	HSIL	Total
Images	801	780	720	2301
C: Test dataset				
	Normal	ASCUS	LSIL	HSIL
Scanning copy	112	47	70	61
Images	157584	250,446		

#### Image enhancement

**Fig. 3:**
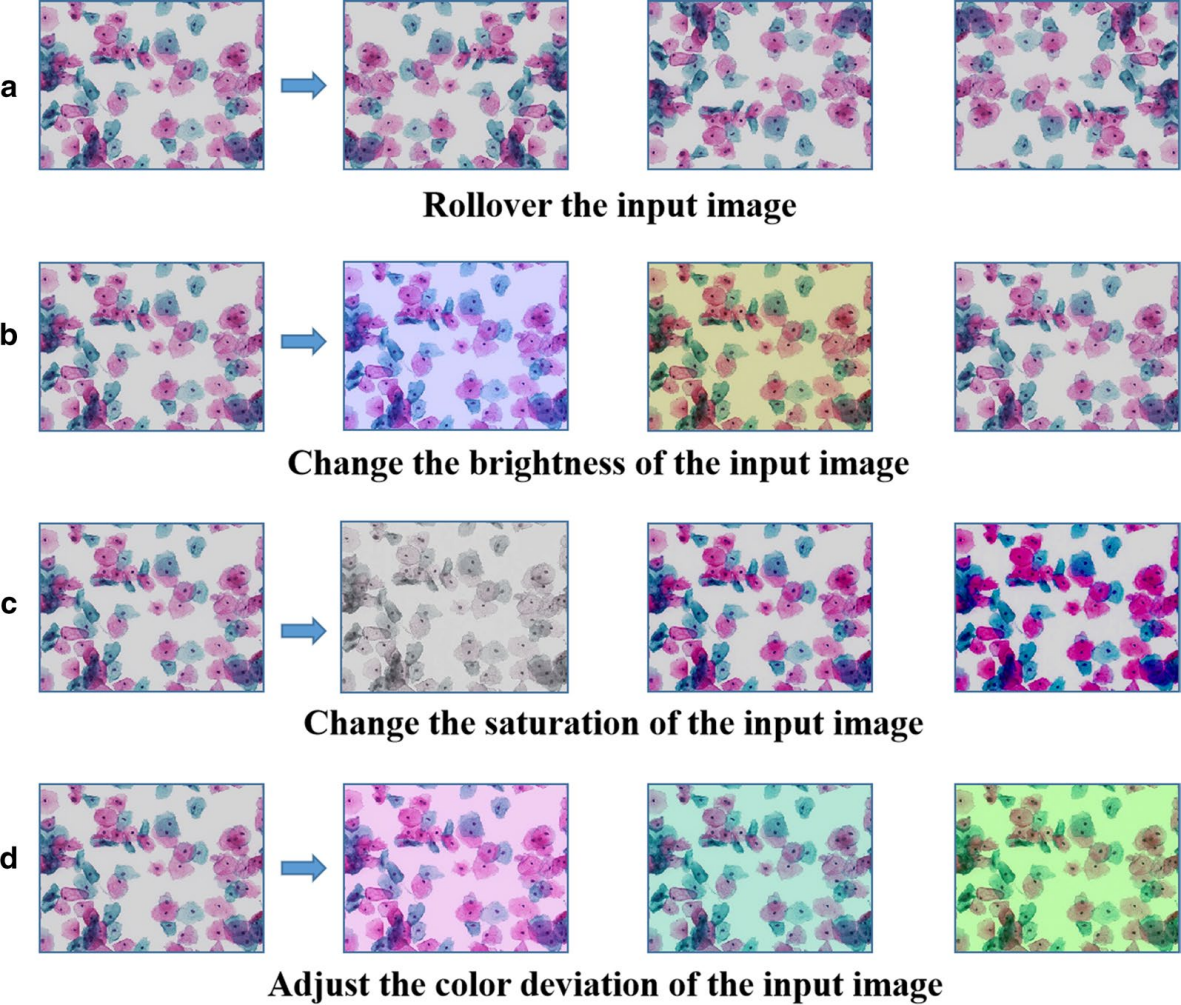
Example image enhancement methods based on color and shape transformations. **a** Image rollover has alternating horizontal and vertical rollover components. Each time, one operation is randomly selected for image processing; **b** Processing of brightness image where the upper limit of the variation coefficient is set to 40, and an integer is randomly selected in the range [0–40] for brightness processing. **c** Processing of the saturation image where the range of the coefficient of change is set to [0.5,2], and the image color space is converted to the HSV space. A random value from the coefficient range is multiplied with the image in the saturation space each time. **d** Processing of the RGB color image where the coefficient of change is set to 60, the three R, G, B component values are randomly selected from [0,60]. In order to increase the image data variability, one or more of the four processing methods is are randomly selected each time

Because the data samples in this study are limited, we employ image processing methods to simulate and transform the color and shape of the sample images in order to increase the data diversity and better simulate the real data variability. The image processing methods were implemented by an image rollover, changing the brightness, saturation, or adjusting the color deviation within the original images. Figure 3 illustrates some of the images that are simulated or transformed during the enhancement process.

### Network model

The processed cervical cytological images, 13,775 pictures, were used as training samples, and the labeled boxes and the pathological changes in irregular cells within the images were used as training labels. The deep CNN architecture contains a convolution kernel, an activation function and a pooling function of 3 × 3 and 1 × 1 sizes. During training, this architecture is used to extract image features and get feature maps. The feature map is fed into the region proposal network, and the IoU metric is used to select the region proposals for real and interfering targets. The proposal box was used to select the feature map in the feature graph of the previous step and process it through the grid pooling layer to obtain a pooled feature map. Then, the real target and interference target features were identified contained in the pooled feature graph by classification network. The deviation of the proposal box and ground-truth box was calculated. The network was optimized by backpropagation combining classification error and proposal box deviation, to achieve higher classification accuracy and more accurate localization of region proposals.

### Loss function

During training, an objective loss function was used to measure the model localization and detection performance. This function is defined as:

1 $$L\left(\left\{\left.{p}_{i}\right\},\left\{\left.{t}_{i}\right\}\right.\right.\right)={\sum }_{i}{L}_{\text{cls}}\left({p}_{i},{p}_{i}^{gt}\right)+{\sum }_{i}{p}_{i}^{gt}{L}_{\text{reg}}\left({t}_{i},{t}_{i}^{gt}\right)$$

where, *i* is the subscript of an anchor in a mini-batch, ${p}_{i}$ is the prediction possibility of an object in anchor *i*, and ${p}_{i}^{gt}$ is the ground-truth tag. If the anchor is positive, the ground-truth label ${p}_{i}^{gt}$ is 1, otherwise, it is 0. ${t}_{i}$ represents four parameterized coordinates of bounding-box of the predicted target region, (anchor-based transformation), ${t}_{i}^{gt}$ is the ground-truth box corresponding to this positive anchor (transformation based on anchor) ${L}_{\text{cls}}$ represents classification loss. ${L}_{\text{reg}}$ stands for regression loss.

#### Regression loss

Regression loss ${L}_{\text{reg}}\left({t}_{i},{t}_{i}^{gt}\right)=R\left({t}_{i},{t}_{i}^{gt}\right)$ is the robust smooth-L1 loss function. ${p}_{i}^{gt}$ indicates that regression loss is only activated when the value of positive anchor (${p}_{i}^{gt}$) is equal to unity. Therefore, the regression adopted the calculation stable smooth-L1 method and used it to calculate the deviation between the predicted target coordinates and ground-truth. Then it minimized the error to make the predicted target position closer to ground-truth to achieve an accurate model positioning. The smooth-L1 function is used to calculate the distance between the coordinates of the predicted and ground-truth targets. The smooth-L1 loss is defined as:

2 $$\text{smooth}-L1\left(\chi \right)=\left\{\begin{array}{c}0\text{.}5{\chi }^{2}\\ \left|\chi \right|-0\text{.}5\end{array}\right.\begin{array}{c}\text{if}\,\left|\chi \right|<1\\ \text{otherwise}\end{array}$$

#### Classification loss

There were two ways to classify the training samples. One was to divide the samples into normal or abnormal ones based on the presence of diseased cells. The classification loss uses cross-entropy, which is a binary classifier (only in the case of yes and no). For each category, the two probabilities predicted by the model were p and 1-p, respectively, and the classification loss is defined as:

3 $${L}_{\text{cls}}\left({p}_{i},{p}_{i}^{gt}\right)=-\text{log}\left[{p}_{i}*{p}_{i}^{gt}+\left(1-{p}_{i}\right)\left(1-{p}_{i}^{gt}\right)\right]$$

The other way was to divide the microscopic field of view into LSIL, HSIL, and ASCUS images based on the lesion degree. For the second classification method, the algorithm encoded multiple categories as a continuous sequence with 0 starting and 1 interval. The classification loss is as follows:

4 $${L}_{\text{cls}}\left({P}_{i},{P}_{i}^{gt}\right)=-{\sum }_{c}^{N}{y}_{c}\bullet \text{log}{p}_{c}$$

where, N represents the number of categories, i.e., the types of lesions, ${y}_{c}$ values 0 or 1, if the sample category is the same as category C, the value is 1, otherwise, it is 0. ${p}_{c}$ represents the probability that the prediction samples belong to category C.

### Model identification

For the feature identification of the same area, the voting method was used to select the category. The number of probability values that each model could output was the same as the number of categories to be identified, i.e., each category corresponded to a probability value. The probability value is directly proportional to the possibility. The higher the probability value is, the higher the possibility value is. The probability outputs of the two models were averaged, and then the category corresponding to the maximum probability value was selected as the output category. The average value calculated by the voting algorithm is as follows:

5 $${P}_{ave}\left(i\right)=\frac{{P}_{{net}_{1}}\left(i\right)+{P}_{{net}_{2}}\left(i\right)+\dots +{P}_{{net}_{m}}\left(i\right)}{m};i=\text{0,1},2,\dots ,N-1$$

where, *i* is the serial number of the category, N is the number of categories to be identified, ${p}_{{\text{net}}_{m}\left(i\right)}$ is the probability value of category i in the N probability values output by the *M* th model, and ${p}_{\text{ave}\left(i\right)}$ is the average probability value of the model in category i, and *m* is the number of models. The category corresponding to the maximum probability value was selected as the final recognition category.

### Statistical analysis

The receiver operating characteristic (ROC) curve was used to evaluate the ability of the auxiliary diagnosis model of cervical cancer to distinguish between the negative and positive images and to calculate the Area Under ROC Curve (AUC) values. AUC is a more objective evaluation index to measure the advantages and disadvantages of the two-classification model. The ROC curve and AUC value are calculated by Python (version 3.6) and scikit -learn (version 0.20.0).

## Results

The samples were divided into normal, ASCUS, LSIL, and HSIL cells as well as squamous cell carcinoma (SqCa). Performance indicators included sensitivity, specificity, positive predictive value (PPV), and negative predictive value (NPV), defined as follows:

6 $$\text{specificity}=\frac{\text{TN}}{\text{TN}+\text{FP}}$$

7 $$\text{sensitivity}=\frac{\text{TP}}{\text{TP}+\text{FN}}$$

8 $$\text{PPV}=\frac{\text{TP}}{\text{TP}+\text{FP}}$$

9 $$\text{NPV}=\frac{\text{TN}}{\text{FN}+\text{TN}}$$

where, TN, TP, FN, and FP represent the numbers of true-negative, true-positive, false- negative, and false- positive images, respectively.

### Evaluation of positive and negative classification

**Fig. 4:**
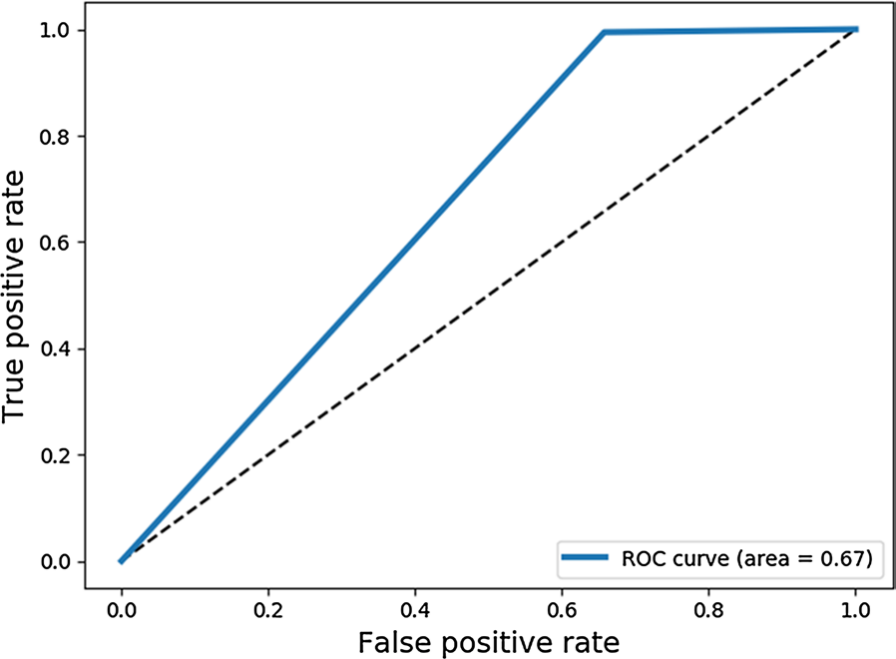
Receiver Operating Characteristic (ROC) curve of the Faster R-CNN system. ROC curve and area under the curve (AUC) of the primary test dataset for the Faster R-CNN system in comparison to the labeling by experienced pathologists

Table 2 shows the confusion matrix of the classification results of the TCT technique used for the test data composed of 290 scans of complete slides. Of the 112 normal slides, 39 were correctly classified as normal and 73 were incorrectly classified as abnormal. Out of 178 abnormal slides, a total of 177 slides were correctly classified. This classifier resulted in a sensitivity of 99.4% and a specificity of 34.8%. The diagnostic test performance was better quantified using the positive predictive value (PPV) and the negative predictive value (NPV). The predictive value represented the probability that an experiment could make a correct judgment and the actual clinical significance of the results. In our system, the PPV and NPV were 70.8 and 97.5%, respectively. Furthermore, we constructed the ROC curve which is a comprehensive indicator of the continuous variables of sensitivity and specificity. The proposed system had an AUC of 0.67 for the ROC curve in Fig. 4.

**Table 2:** Diagnostic performance in classifying normal and abnormal cervical cells in the test dataset

CNN system	Turth		Total
	Normal	Abnormal	
Normal	177 (TP)	73 (FP)	242
Abnormal	1 (FN)	39 (TN)	48
Total	178	112	290

### Evaluation of lesiondegree classification

Our tests were performed on 1975 images generated after slide segmentation as shown in Fig. 2. This system didn’t provide a direct diagnosis but provided a reference for the pathologists according to the cells with the highest degree of the lesion. Table 3 shows the confusion matrix used to test the results of TCT classification according to lesion degree. Of the 606 ASCUS images, 541 were correctly classified. Also, 422 of the 590 LSIL images and 576 of the 799 HSIL images were classified correctly. Therefore, the sensitivities for the ASCUS, LSIL, and HSIL were 89.3, 71.5, and 73.9%, respectively.

**Table 3:** Performance metrics for the Faster R-CNN model, assessed on single filed images selected from the test dataset

	ASCUS	LISL	HSIL
True-positive	541	422	576
False-negative	65	168	193
Total	606	590	779
Sensitivity	89.3%	71.5%	73.9%

## Discussion

In this paper, we have proposed a cervical cancer screening method based on CNN, which could successfully extract features from TCT images. The pathological condition of glass slides was diagnosed by dividing the images scanned by glass slides into a large number of single visual field images to establish the model, and by integrating the results of the visual field processing. In general, this model could distinguish between negative and positive cells, with a recognition sensitivity of 99.4%. Proper diversion of negative and positive marker images was also the focus of developing the cervical cancer-assisted screening system. This model achieved a 99.6% accuracy to exclude negative images (i.e. normal or healthy populations), and the remaining suspicious images were analyzed and confirmed by pathologists.

The specificity of the system was only 34.8%, and hence many normal cells were misclassified as abnormal ones. However, the key goal of this model was to ensure that there were no false-negative images as much as possible, and thus reduce the rate of missed diagnosis. The PPV was 70.8%, which meant that the test-positive patients had a 70.8% chance of actually having the disease. The NPV was 97.5%, which meant that the test-negative patients had a 97.5% chance of actually not having the disease. Although the low specificity means that more than half of the normal women may be classified as suspicious cases for further diagnosis, the system had a low false-negative rate of 0.06%, which indicated that the rate of missed diagnosis greatly decreased and it could effectively identify suspicious cases. This also helps pathologists improve their disease diagnosis efficiency, so they can spend more time on analyzing complex cases and ensuring that diseases are diagnosed quickly and accurately. Although cervical cancer screening has become very popular, there is an imbalance in medical resources and untrained pathologists in some parts of China. Our deep learning model may help solve the above problems, and lead to a more accurate and effective diagnosis.

Moreover, this model can be re-classified according to the abnormal cell details. The sensitivity of ASCUS, LSIL, and HSIL was 89.3, 71.5, and 73.9%, respectively. According to the degree of a lesion, the classification of cervical cancer cells indicated that the model has good sensitivity to identify the degree of a lesion in a single field. The diagnosis of the TCT smear is a synthesis of the results of a large number of the single-field images, and our system was able to assist the specialists in locating and identifying different degrees of diseased cells quickly, allowing them to devote more time on more complex cases.

To our knowledge, only few studies on automatic diagnosis of cervical cancer were based on ThinPrep cytologic test (TCT) images instead of pap smears. This is mainly because TCT images are relatively difficult to analyze compared to the pap smear images. More specifically: (1) the TCT images do not currently have a good database, and the collection of such an image database is more difficult compared to the Harlve database of pap-smear images [38, 39]; (2) TCT images have many overlapping cells, which are not as easy to analyze as the single cells in pap-smear images [40, 41]; (3) the color and quality of TCT images obtained from different medical institutions may vary greatly. Therefore, these systems are poorly adapted for the proposed system, we trained it with data from hospitals at different levels in different regions of north and south China, which ensured the high adaptability of the system. Theoretically, the CNN system has good robustness and low dependence on smears staining and preparation. The system can adapt to the images from different hospitals and reduce the secondary examination caused by low-quality images. Future research is needed for further verification in other clinical applications.

A sensitivity screening system may help pathologists to quickly diagnose some positive lesions with a computational speed of 70 − 10 milliseconds per single field. Therefore, this system can relieve the burden of the pathologists to some extent. In addition, the system not only reduces the influence of appellate factors, and ensures consistency in diagnosing images of different pathologists from different hospitals, but also increases the possibility of remote diagnosis. Meanwhile, because the automatic cervical cancer diagnosis system is mainly based on CNN models, the construction and diagnosis costs are very low.

However, our proposed system also has several major limitations. Firstly, Due to a high number of overlapping and adhesion cells, the differences between diseased cells and normal cells were not distinct. It was difficult for the system to learn the characteristics of cells, and hence its accuracy was relatively low. Secondly, the collected dataset didn’t have enough samples of some pathological types such as atypical squamous cells-cannot exclude high-grade squamous intraepithelial lesion (ASC-H) or atypical glandular cells (AGC). This scarcity can’t lead to effective training and accurate classification. Therefore, we plan to collect more samples and enlarge our dataset in order to arrive at more valuable findings. Ultimately, it is difficult to obtain complete TCT image information from outpatients. Future research should focus on more relevant patient information, such as age, HPV infection, or vaginal inflammation. Similarly, large amounts of data will be needed to design prospective follow-up studies to enhance the stability and accuracy of the model. In addition, with the discovery of new biomarkers, tumor diagnosis based on molecular imaging may be explored as a viable path [42–44].

## Conclusions

The CNN-based TCT cervical cancer cell classification system proposed in this paper can effectively exclude negative smear samples, and identify the suspicious population in cervical cancer screening. It also showed high sensitivity and excellent performance in the identification of cervical cancer screening, which can save time for pathologists and provide an excellent secondary prevention effect. In comparison to the conventional diagnostic methods, this system has good robustness, objectivity, and small computational cost. Meanwhile, our system provides a possibility for online diagnosis of TCT images and is expected to contribute to the construction of primary medical care.

## Data Availability

All the data used in this article is available from the corresponding authors on reasonable request.

## Abbreviations

HPV: Human papillomavirus
TCT: Thinprep Cytologic Test
Faster R-CNN: Faster region- convolution neural network
NCCN: National Comprehensive Cancer Network
DCNN: Deep convolutional neural network
FDA: Food and Drug Administration
ANN: Artificial neural network
ASCUS: Atypical squamous cells of undetermined significance
NILM: Intraepithelial Lesion/Malignant Lesion cell
ASC-H: Atypical squamous cells-cannot exclude high-grade squamous intraepithelial lesion
AGC: Atypical glandular cells
LSIL: Low-grade squamous intraepithelial lesion
HSIL: High grade squamous intraepithelial lesions
SqCa: Squamous cell carcinoma
CT: Computerized tomography
MRI: Magnetic Resonance Imaging
AUC: Area under the curve
ROC: Receiver operating characteristic
TN: Ture negative
TP: Ture positive
FN: False negative
FP: False positive
PPV: Positive predictive value
NPV: Negative predictive value
